# The clinical efficacy of combined ESA and Roxadustat treatment for renal anemia in hemodialysis patients with secondary hyperparathyroidism: A case series

**DOI:** 10.1097/MD.0000000000039083

**Published:** 2024-08-16

**Authors:** Jing-jing Zhong, Ming-li Wang, Gao-feng Zheng, Ming-peng Li, De-zheng Chen

**Affiliations:** a Department of pharmacy, The People’s Hospital of JianYang City, Jianyang, Sichuan, China.

**Keywords:** erythropoiesis stimulators (ESAs), hemodialysis, hyperparathyroidism, renal anemia, Roxadustat

## Abstract

**Rationale:**

Pharmacological mechanism of Roxadustat in the treatment of renal anemia.

**Patient concerns:**

To investigate the efficacy and safety of combined Roxadustat and erythropoiesis stimulator (ESA) treatment of renal anemia in hemodialysis patients with secondary hyperparathyroidism.

**Diagnoses:**

A retrospective analysis was conducted on hemodialysis patients with renal anemia and secondary hyperparathyroidism treated with ESAs alone, who were admitted to our hospital from March 2022 to December 2022.

**Interventions:**

The patients were treated with Roxadustat combined with ESAs for 3 months, during which oral iron supplementation was given, and the changes in Hb levels and laboratory-related indicators before and after the combined treatment were analyzed.

**Outcomes:**

The results showed that a total of 13 patients received combination therapy, with a significant increase in Hb compared to ESAs alone (*t* = −3.955, *P* = .002). The Hb qualification rate was 38.46%, and the ∆Hb response rate was 76.92%. The parathyroid hormone significantly decreased with a statistically significant difference (*Z* = −2.062^b^, *P* = .039). Hemoglobin (RBC), total iron binding capacity, and serum ferritin (male) were significantly increased compared to ESAs alone. Total cholesterol and low-density lipoprotein were significantly lower than ESAs alone. The differences in the changes in the above indicators were statistically significant (*P* < .05). There was no statistically significant difference in changes in other laboratory-related indicators (*P* > .05). No adverse reactions were observed during the combined treatment of 13 patients.

**Lessons subsections:**

The combination of Roxadustat and ESAs can effectively improve renal anemia in hemodialysis patients with secondary hyperparathyroidism, as well as improve indicators of hyperparathyroidism and blood lipid levels with high levels of safety. This combined treatment thus provides a new and safe treatment method for these patients.

## 1. Introduction

Renal anemia is a common complication of treatment for chronic kidney disease (CKD). Anemia not only adversely affects the quality of life of patients with kidney disease but also accelerates disease progression, increasing the risk of cardiovascular events and death.^[1]^ At present, the main drugs used for the clinical treatment of renal anemia include erythropoiesis-stimulating agents (ESAs), iron supplements, and hypoxia-inducible factor prolyl hydroxylase inhibitors (HIF-PHIs). Iron supplements can cause adverse gastrointestinal reactions such as constipation and nausea, and may even lead to severe life-threatening allergic reactions, and thus their application is limited. HIF-PHIs are new therapeutic agents and have not yet been widely used in clinical practice due to various factors, including cost. ESAs are still the most commonly used drugs for the clinical treatment of renal anemia but the simple administration of ESAs often does not achieve satisfactory results, and about 5% to 10% of patients respond poorly to ESAs.^[2]^ Research has found that the main reasons for the poor efficacy of ESAs include the presence of iron deficiency, inflammation, erythropoietin (EPO) inhibitors, malignant tumors, and secondary hyperparathyroidism.^[3,4]^

Roxadustat is a representative HIF-PHI and can upregulate the expression of EPO and the EPO receptor, as well as genes involved in the regulation of iron metabolism, by modulating the expression of HIF and its downstream targets, ultimately enhancing erythropoiesis. The current study investigated the efficacy of Roxadustat in a subgroup of Chinese ESA-resistant hemodialysis patients with renal anemia. It was found that among patients who had received stable therapeutic doses of ESAs before enrollment and had baseline Hb < 100 g/L before changing to treatment with Roxadustat for 28 weeks, 94.4% had Hb ≥ 100 g/L and 83.3% of patients had Hb ≥ 110 g/L^[5]^; Similar results were also obtained in the subgroup analysis of an ESA-resistant population of hemodialysis patients in Japan.^[6]^ It has been reported that ESAs combined with Roxadustat can effectively corrects ESA-hyporesponsive anemia in patients on peritoneal dialysis.^[7]^ In addition, Roxadustat has been found to improve both iron metabolism^[8]^ and micro-inflammation in patients,^[5,9–11]^ which may be associated with the mechanism underlying the effects of Roxadustat on renal anemia in dialysis patients who respond poorly to ESA-only treatment.

Another common complication in hemodialysis patients with CKD is secondary hyperparathyroidism. This often coexists with renal anemia, and the severity of anemia is negatively correlated with the levels of parathyroid hormone (iPTH).^[12]^ This may be related to the effects of PTH on bone marrow hematopoiesis, which influences the synthesis of endogenous EPO and shortens the lifespan of red blood cells.^[13]^ The poor therapeutic effect of ESAs is reported to be directly associated with increased PTH levels, with high PTH levels significantly inhibiting the clinical efficacy of ESAs and necessitating increased dosages.^[14]^ Based on the pharmacological action of Roxadustat in upregulating the expression of endogenous EPO, we hypothesized that Roxadustat may be effective for treating renal anemia hemodialysis patients with secondary hyperparathyroidism who respond poorly to ESAs alone.

This study used a retrospective analysis to evaluate the efficacy and safety of Roxadustat used in combination with ESAs for the treatment of renal anemia in the above patients, providing new ideas for the treatment of such patients.

## 2. Methods

### 2.1. Research participants

A retrospective analysis was conducted on hemodialysis patients with renal anemia and secondary hyperparathyroidism treated with ESAs alone from March 2022 to December 2022 in our hospital. The inclusion criteria were: (1) age ≥ 18 years; (2) hemodialysis patients with renal anemia who had shown a poor response to ESA treatment, specifically, those who had received previous regular subcutaneous injections of recombinant human erythropoietin (rHuEPO of 18,000 U/week) and whose hemoglobin (Hb) levels were <100 g/L after 4 months of treatment; (3) the presence of secondary hyperparathyroidism; (4) no intravenous iron supplementation during treatment, although oral iron supplementation was allowed; (5) complete clinical data. The exclusion criteria were: (1) recent experience of active bleeding diseases; (2) anemia caused by other reasons; (3) malignant hypertension; (4) tumor; (5) stroke; (6) patients with a history of severe drug allergy or known allergy to the active ingredients or excipients of Roxadustat. In addition, patients were excluded if the enrolled patients had any of the above exclusion criteria or died during the observation period. The study was approved by the Ethics Committee of Jianyang People’s Hospital. All enrolled patients provided written informed consent.

### 2.2. Therapeutic method

After previous regular use of rHuEPO did not meet the standard (Hb < 100 g/L), treatment was changed to combination therapy and oral Roxadustat treatment. The starting dose of Roxadustat was 100 mg (<60 kg body weight) or 120 mg (≥60 kg body weight) for each dose, administered orally, 3 times a week. During the initial treatment stage, Hb levels were monitored every 2 to 4 weeks, with adjustment of the Roxadustat dose according to the Hb level. Treatment was continued and the patients were monitored for 12 weeks.

For the primary disease, concomitant disease, or comorbidities associated with chronic renal failure, such as hypertension, diabetes, disorders of bone mineral metabolism, ion disorders or acidosis, volume overload, or cardiovascular and cerebrovascular comorbidities, treatment was continued and the medication was adjusted according to the corresponding guidelines or consensus. None of the patients received intravenous iron supplementation during the treatment but were allowed oral iron therapy. The dose of iron supplementation was 100 to 300 mg/d (elemental iron). Patients with hyperphosphatemia (pre-dialysis blood phosphorus ≥ 1.45 mmol/L) were treated with non-calcium-containing phosphorus binders (lanthanum carbonate chewable tablets or sevelamer hydrochloride) to reduce phosphorus levels.

The dosage of cacimimetics, sevelam, lanthanum carbonate, vitamin D, and iron varies according to the patient’s condition, and the conventional dosage is 25 mg qd, 1600 mg tid, 500 mg tid, 0.5 μg qd, 0.3 g qd, respectively. Before and after the intervention, the use of the above drugs would be adjusted appropriately with the blood calcium, blood phosphorus and iron reserves of patients, and try to maintain these indicators to reach normal values.

Letters of comfort were issued by the Ethics Committee of Jianyang City People’s Hospital. All methods were carried out in accordance with the ethical principles of the Declaration of Helsinki and the Methods for the Ethical Review of Biomedical Research Involving Humans issued by the National Health and Family Planning Commission of China. All experimental protocols were approved by the Ethics Committee of Jianyang City People’s Hospital (ID: JY202216). Ethics Committee of Jianyang City People’s Hospital waived the need of informed consent since the study did not involve personal privacy, any further invasive interventions, or commercial interests.

### 2.3. Outcome measures

The following indicators were recorded before and after the addition of Roxadustat to the treatment:

1. Indicators of anemia: Hemoglobin (Hb), red blood cell count (RBC), serum ferritin (SF), total iron-binding capacity (TIBC), and transferrin saturation (TSAT).
2. Indicators of parathyroid function: Blood levels of calcium, phosphorus, potassium, alkaline phosphatase (ALP), serum creatinine (SCr), urea nitrogen, and iPTH.
3. Inflammation: White blood cell count (WBC), neutrophil count (NEUT), and lymphocyte count (LYM).
4. Blood lipids and blood glucose: Low-density lipoprotein cholesterol (LDL-C), high-density lipoprotein cholesterol (HDL-C), total cholesterol (TC), triglycerides (TG), and random blood glucose.
5. Blood pressure: Systolic and diastolic blood pressure.

The endpoint for efficacy assessment was the alleviation of anemia. The main outcome was the change in Hb level (ΔHb). The secondary outcomes were the Hb response rate and the Hb qualification rate. The Hb response rate was defined as the proportion of participants who achieved ΔHb values above 10 g/L after 3 months of treatment. The Hb qualification rate was the proportion of participants whose Hb level reached 110 g/L at the end of the 3-month treatment. Adverse events were recorded for the evaluation of safety endpoints.

### 2.4. Statistics

SPSS 22.0 software was used for data analysis. The measurement data of Hb, RBC, WBC, NEUT, LYM, blood calcium, blood phosphorus, blood potassium, SCr, urea nitrogen, ALP, LDL-C, HDL-C, TC, TG, random blood glucose, systolic blood pressure, and diastolic blood pressure that conformed to a normal distribution are expressed as means and standard deviations χ̄ ± s and paired sample *t* tests were used for comparisons between single groups before and after treatment. The non-normally distributed measurement data, including SF, TIBC, TSAT, and iPTH, are expressed in the form of M (P_25_, P_75_), and Wilcoxon rank-sum tests for paired samples were used for comparisons between the 2 groups. *P* < .05 was considered statistically significant.

This paper used the STROBE cross-sectional reporting guidelines, such as: von Elm E, Altman DG, Egger M, Pocock SJ, Gotzsche PC, Vandenbroucke JP. The Strengthening the Reporting of Observational Studies in Epidemiology (STROBE) Statement: guidelines for reporting observational studies.

## 3. Results

### 3.1. Basic information on patients

A total of 13 eligible patients were enrolled, including 3 males and 8 females, with an average age of 50.54 ± 11.59 years and an average dialysis duration of 29.85 ± 16.07 months. The primary diseases of the patients were 7 cases of chronic nephritis syndrome, 2 cases of diabetic nephropathy, 1 cases of Henoch Schonlein purpura nephritis, 1 case of anti-GBM nephritis, 1 case of crescentic nephritis, and 1 case of renal damage associated with systemic vasculitis.

### 3.2. Hb levels before and after combination therapy

The Hb levels of the 13 patients after treatment with the combination of Roxadustat and rHuEPO were significantly higher than after treatment with rHuEPO alone. When rHuEPO was used alone, the baseline Hb level was 77.46 ± 11.32 g/L, while after 3 months of the combined treatment the mean Hb level was 101.23 ± 20.76 g/L, the Hb qualification rate was 38.46% (5/13), and the ∆Hb response rate was 76.92% (10/13). The change in Hb level with the combined treatment was statistically significant (*t* = −3.955, *P* = .002) (Table 1).

**Table 1:** Indicators showing significant differences between the ESA-only and ESA + Roxadustat treatments.

Index type	Clinical indicator	Baseline level	After 3 months of treatment	Statistic (*t*/*Z*)	*P* value
Anemia index	Hb g/L	77.46 ± 11.32	101.23 ± 20.76	−3.955	.002
	RBC 10^12^/L	2.80 ± 0.54	3.78 ± 0.83	−4.882	<.001
	TIBC μmol/L	49.63 ± 11.01	58.41 ± 10.68	−2.726^b^	.006
	SF (Male) ng/mL	57.71 ± 20.13	32.00 ± 13.43	−2.023^b^	.043
Parathyroid function index	iPTH pg/mL	393.66 ± 405.54	200.56 ± 108.12	−2.062^b^	.039
Blood lipids	TC mmol/L	3.54 ± 0.68	2.86 ± 1.08	3.639	.003
	LDL-C mmol/L	1.82 ± 0.51	1.48 ± 0.88	−2.272^b^	.023

Hb = hemoglobin, iPTH = parathyroid hormone, LDL-C = low-density lipoprotein cholesterol, SF = serum ferritin, TC = total cholesterol, TIBC = total iron-binding capacity.

### 3.3. Changes in iPTH levels before and after the combined treatment

The iPTH levels of the 13 patients treated with Roxadustat combined with rHuEPO were significantly lower than those with rHuEPO treatment alone. The baseline iPTH level was 393.66 ± 405.54 pg/mL with rHuEPO alone, while after 3 months of treatment with Roxadustat, the level was reduced to 200.56 ± 108.12 pg/mL, showing a statistically significant change (*Z* = −2.062^b^, *P* = .039) (Table 1).

### 3.4. Changes in other indicators before and after the combination therapy

The RBC, TIBC, and SF (male) anemia indicators in the 13 patients after combined treatment with Roxadustat and rHuEPO were significantly higher than those treated with rHuEPO alone. Compared with rHuEPO treatment alone, the levels of both TC and LDL-C were significantly lower; these changes were statistically significant (*P* < .05) (Table 1). There were no significant differences in other indicators after Roxadustat treatment (*P* > .05) (Table 2).

**Table 2:** Indicators showing no significant differences between the ESA-only and ESA + Roxadustat treatments.

Index type	Clinical indicator	Baseline level	After 3 months of treatment	Statistic (*t*/*Z*)	*P* value
Anemia index	SF (Female) ng/mL	94.83 ± 76.57	71.81 ± 54.58	−1.120^b^	.263
	TSAT %	20.38 ± 7.44	17.74 ± 9.48	−1.782^b^	.075
Parathyroid function index	Blood calcium mmol/L	2.20 ± 0.21	2.33 ± 0.19	−1.992	.070
	Blood phosphorus mmol/L	2.05 ± 0.74	1.89 ± 0.57	0.639	.535
	Blood potassium mmol/L	4.70 ± 0.59	4.78 ± 0.71	−0.381	.710
	ALP U/L	83.85 ± 23.75	80.62 ± 16.66	0.674	.513
	SCr μmol/L	1047.85 ± 271.96	1019.46 ± 261.82	−0.454^b^	.650
	Urea nitrogen mmol/L	27.68 ± 11.30	24.60 ± 7.04	1.088	.298
Inflammatory index	WBC 10^9^/L	6.12 ± 1.94	6.88 ± 2.43	−1.712^b^	.087
	NEUT 10^9^/L	4.53 ± 1.78	5.19 ± 2.36	−1.782^b^	.075
	LYM 10^9^/L	1.04 ± 0.31	1.03 ± 0.24	0.200	.845
Blood sugar	Random blood glucose mmol/L	6.64 ± 1.49	6.57 ± 2.07	−0.128	.901
Blood lipids	TG mmol/L	1.54 ± 0.88	1.42 ± 0.69	−0.350^b^	.727
	HDL-C mmol/L	1.10 ± 0.28	0.96 ± 0.31	−1.958^b^	.0502
Blood pressure	Systolic pressure mm Hg	136.08 ± 21.23	138.08 ± 22.29	−0.355	.729
	Diastolic pressure mm Hg	80.92 ± 13.17	81.15 ± 12.58	−0.063	.951

ALP = alkaline phosphatase, HDL-C = high-density lipoprotein cholesterol, LYM = lymphocyte count, NEUT = neutrophil count, SCr = serum creatinine, SF = serum ferritin, TG = triglycerides, TSAT = transferrin saturation, WBC = white blood cell count.

### 3.5. Adverse events

An analysis of the incidence of adverse events before and after the combination therapy showed that there were no significant gastrointestinal reactions in the 13 patients treated with Roxadustat combined with rHuEPO during the observation period, nor were there any serious adverse reactions such as significantly aggravated hypertension, new deep vein thromboembolism, damaged liver function, hyperkalemia, myocardial infarction, or heart failure.

## 4. Discussion

The clinical practice guidelines for the diagnosis and treatment of renal anemia in China specify using the Hb levels as the standard for both diagnosing and evaluating the effects of treatment of renal anemia, while SF, TSAT, and TIBC are recommended as auxiliary diagnostic criteria for the presence of dystrophic anemia. In this study, previous regular use of rHuEPO had failed to raise the Hb levels to meet the standard in 13 patients before the addition of Roxadustat to the treatment. After 3 months of treatment with Roxadustat, the Hb levels of the patients had increased to varying degrees, with a compliance rate of 38.46% (5/13). Considering that the baseline Hb levels of these patients were low and there were many factors influencing renal anemia, although the Hb levels of 8 patients were still not up to standard, they had increased significantly to 23.77 ± 21.67 g/L after only 3 months of the combined treatment. Among the patients, the Hb levels of ten patients increased by more than 10 g/L in 3 months, with the highest increase of 62 g/L, indicating that rHuEPO combined with Roxadustat was more effective than rHuEPO alone in the treatment of renal anemia in patients with hyperparathyroidism and that it can take effect within 3 months. Changes were also seen in the RBC, SF, TSAT, and TIBC anemia indicators. RBC, TIBC, and SF (male) increased significantly after 3 months of the combined treatment, while SF (female) and TSAT did not change significantly. This may be due to the average SF and TSAT values being close to normal before starting the combined treatment, and the change in SF was related to sex. These results show that the combination of Roxadustat with rHuEPO can alleviate renal anemia mainly by increasing the Hb, RBC, TIBC, and SF levels. As mentioned in ^[Section 1]^ , Roxadustat can increase the production of red blood cells by upregulating the expression of EPO, among other mechanisms. The present study also confirmed that the combined use of Roxadustat and ESA treatment in patients whose Hb and RBC levels had not improved after 4 months of continuous use of rHuEPO could reverse this situation. Despite the failure of the Hb and RBC levels to meet the standard in some patients, this was related to the serious anemia of patients and the short duration of Roxadustat treatment.

Parathyroid hormone can affect the release of EPO both directly and indirectly,^[15]^ as well as interfering with erythropoiesis,^[16]^ shortening the lifespan of erythrocytes,^[17]^ and reducing the sensitivity of peripheral tissues to EPO.^[16]^ In this study, 13 patients were treated with Roxadustat combined with rHuEPO for 3 months, after which the hormone levels were significantly lower than in patients treated with rHuEPO alone, indicating that the improvements in renal anemia observed in the patients in this study occurred through the reduction of iPTH hormone production by Roxadustat, leading to increased expression of endogenous EPO and altering the levels of Hb, RBC, and TIBC, and finally leading to the improvement of renal anemia in patients. However, Roxadustat did not affect parathyroid function through blood calcium, blood phosphorus, blood potassium, serum ALP, SCr, or urea nitrogen.

Dyslipidemia is very common in hemodialysis patients with parathyroid dysfunction, and also promotes the progression of CKD. It is associated with an increased risk of cardiovascular events,^[18,19]^ and is mainly manifested by high TG, low HDL-C levels, and variable levels of LDL-C and TC.^[20]^ Statins are the first-line drugs for the treatment of patients with cardiovascular disease and CKD.^[17]^ A clinical trial of the most common lipid-lowering drugs for patients with CKD included in the heart and kidney protection study (SHARP) showed that compared with placebo, the risk of atherosclerotic cardiovascular disease in patients treated with statins decreased by 17%, but in the 4.9-year follow-up, 11.3% of patients in the treatment group had still experienced major cardiovascular events.^[21]^ Roxadustat is a new drug developed for the treatment of renal anemia. In addition to effectively controlling anemia, its effects on blood lipids have also received attention. Both foreign phase II and domestic phase II and phase III clinical trials showed that the levels of TC and LDL-C were significantly reduced after Roxadustat treatment in both nondialysis and dialysis patients, but there was no change in patients receiving EPO treatment.^[22,23]^ At present, the mechanism by which Roxadustat reduces blood lipid levels is not clear. HIF is the main transcription factor that regulates adaptation to hypoxia, through controlling the transcription of more than 1000 target genes that participate in a wide range of pathophysiological processes. Hwang et al^[24]^ showed that HIF-α can reduce the activity of 3-hydroxy-3-methyl-glutaryl coenzyme A reductase and ultimately reduce cholesterol synthesis in the liver. Zhang et al^[25]^ also showed that Roxadustat could upregulate HIF-2-α in adipocytes, increasing both the excretion of liver cholesterol and reducing the plasma cholesterol level, and thus having a protective effect on atherosclerosis. It was also found that Roxadustat was effective in treating obesity-induced atherosclerosis. The above studies offer suggestions for the mechanism underlying the lipid-reducing effects of Roxadustat. In the present study, we observed that combined treatment with Roxadustat could effectively reduce the levels of TC and LDL-C in patients compared with rHuEPO alone but had little effect on TG. This shows that Roxadustat is effective for modulating lipid metabolism in patients with hyperparathyroidism, especially in patients who respond poorly to statin treatment, with no adverse reactions such as major cardiovascular events observed, which may provide additional benefits to such patients.

The study has several limitations. First, the observation period of 3 months was not sufficient to collect data on drug-induced adverse events. Second, a retrospective analysis cannot confirm causal relationships between drugs and events. In this case, we did not provide an obvious adverse drug reaction profile. Prospective studies will be designed in the follow-up to collect data on possible adverse reactions. At last, because hemodialysis patients must be maximized on IV iron before moving to ESA and other agents. However, in this study, our subjects preferred oral iron. There are common adverse effects of intravenous iron therapy, such as increased risk of infection and cardiovascular events, and even life-threatening hypersensitivity reactions, especially in older patients with a higher incidence of renal anemia. This makes the clinical for such patients have to choose a safer oral iron supplement, but the effect of iron supplementation is often unsatisfactory. Studies have reported that Roxadustat can also maintain Hb above the target value in the treatment of renal anemia in calcification defense dialysis patients with oral iron supplementation.^[26]^ Therefore, through this retrospective study, we want to confirm that Roxadustat combined with ESAs can be used to treat hemodialysis patients with renal anemia accompanied by secondary hyperparathyroidism, which can avoid serious adverse reactions caused by intravenous iron supplementation.

## 5. Conclusions

In conclusion, a combination of EPO and Roxadustat treatment was found to effectively alleviate renal anemia in hemodialysis patients with secondary hyperparathyroidism. Furthermore, this combined treatment also improved hyperparathyroidism and blood lipid levels in patients, with high safety, thus providing a new and safe treatment for such patients. However, due to the retrospective nature of the study, the relatively small number of cases, and the short observation time, further investigation is required for verification of the findings. In the next step, this research group will design relevant prospective cohort studies for confirmation of the above findings.

## Acknowledgments

The authors would like to thank all the reviewers who participated in the review, as well as MJEditor (www.mjeditor.com) for providing English editing services during the preparation of this manuscript and Ran Z-X for the statistical consultation on the data in this article.

## Author contributions

**Project administration:** Jing-jing Zhong, Ming-li Wang, Gao-feng Zheng, De-zheng Chen.

**Writing – original draft:** Jing-jing Zhong.

**Writing – review & editing:** Jing-jing Zhong, Ming-li Wang.

**Investigation:** Ming-li Wang, Ming-peng Li, De-zheng Chen.

**Formal analysis:** Gao-feng Zheng.

**Supervision:** Gao-feng Zheng.

**Data curation:** Ming-peng Li.
